# C1q ablation exacerbates amyloid deposition: A study in a transgenic mouse model of ATTRV30M amyloid neuropathy

**DOI:** 10.1371/journal.pone.0175767

**Published:** 2017-04-13

**Authors:** Elena Panayiotou, Eleni Fella, Revekka Papacharalambous, Stavros Malas, Maria Joao Saraiva, Theodoros Kyriakides

**Affiliations:** 1 Clinic A, Neuropathology Department, The Cyprus Institute of Neurology & Genetics, Nicosia, Cyprus; 2 The Cyprus School of Molecular Medicine, Nicosia, Cyprus; 3 Instituto de Inovação e Investigação em Saúde (I3S) and Neurobiologia Molecular-Instituto de Biologia Molecular (IBMC) - Universidade do Porto, Porto, Portugal; Semmelweis Egyetem, HUNGARY

## Abstract

ATTRV30M amyloid neuropathy is a lethal autosomal dominant sensorimotor and autonomic neuropathy, caused by deposition of amyloid fibrils composed of aberrant transthyretin (TTR). Ages of onset and penetrance exhibit great variability and genetic factors have been implicated. Complement activation co-localizes with amyloid deposits in amyloidotic neuropathy and is possibly involved in the kinetics of amyloidogenesis. A candidate gene approach has recently identified C1q polymorphisms to correlate with disease onset in a Cypriot cohort of patients with ATTRV30M amyloid neuropathy. In the current study we use a double transgenic mouse model of ATTRV30M amyloid neuropathy in which C1q is ablated to elucidate further a possible modifier role for C1q. Amyloid deposition is found to be increased by 60% in the absence of C1q. Significant up regulation is also recorded in apoptotic and cellular stress markers reflecting extracellular toxicity of pre-fibrillar and fibrillar TTR. Our data further indicate that in the absence of C1q there is marked reduction of macrophages in association with amyloid deposits and thus less effective phagocytosis of TTR.

## Introduction

Hereditary ATTRV30M amyloidosis is a lethal autosomal dominant sensorimotor and autonomic neuropathy due to the deposition of amyloid fibrils, in which the main polypeptide is aberrant transthyretin (TTR). In this neuropathy TTR has a substitution of methionine for valine at position 30 of the protein [1]. TTR is primarily produced in the liver (95%) while some is also produced by the choroid plexus and retina. ATTRV30M amyloidosis was first described by Andrade in 1952 in Northern Portugal and other major foci have subsequently been described in Sweden and Japan [2–4]. This mutation is the most common neuropathic mutation among over more than a 100 amyloidogenic point mutations identified worldwide in the TTR gene. TTR circulates in the plasma as a tetramer, however mutant TTR has a higher propensity to dissociate into monomers which misfold and get deposited in various tissues forming amyloid deposits [5].

Penetrance and age of onset of ATTRV30M amyloidotic polyneuropathy varies significantly among different populations. Penetrance in Sweden, Cyprus and Portugal are 2 to 22%, 28% and 80%, while the age of onset is 52, 46 and 32 years of age respectively [6, 7]. Genetic and epigenetic factors have been speculated to play a role. In a Cypriot cohort of patients we have previously demonstrated a correlation between the age of onset of disease and C1q polymorphisms suggesting that C1q may be a genetic modifier [8].

Immunohistochemical examination of amyloid deposits in sural nerve biopsies reveals co-aggregation of TTR with several other proteins, including apolipoprotein E, serum amyloid P and complement C1q [9].

In other amyloidoses such as Alzheimer disease, C1q has been shown to modulate beta-amyloid induced complement activation and neuronal loss [10]. On the other hand C1q has been shown to be neuroprotective against toxic concentrations of serum amyloid P, to modulate phagocytosis of soluble pre-amyloid aggregates and to bind to apoptotic cells and cellular debris [11, 12]. Thus, C1q probably plays a generic role in the pathogenesis of amyloidoses and may be a candidate as a modifier in the phenotype of ATTRV30M neuropathy.

The objective of the current study was to evaluate the role of C1q in the ATTRV30M neuropathy mouse model of the disease, the mTTR^-/-^hMet30^+/+^ mouse, which replicates amyloid distribution seen in this disease with the exception of lack of peripheral nerve involvement [13]. We set out to test the hypothesis that complement C1q is a disease modifier in this animal model.

The ATTRV30M mouse model of the disease (mTTR^-/-^hTTR^MET30+/+)^ was cross bred with a mouse strain lacking C1q (mC1q^-/-^) thus giving rise to a strain deficient in both mouse TTR and mouse C1q but expressing the human mutated TTR transgene. The C1q deficient mice were found to exhibit a 60% increase in amyloid deposition, compared to the C1q efficient mice, and this was associated with a reduced recruitment of macrophages at the site of amyloid deposits and presumably reduced phagocytosis.

## Materials and methods

### Animals and tissue handling

The C1q ablated ATTRV30M mice were derived from the mTTR^-/-^hTTR^Met30+/+^ line previously published (Kohno et al., 1997). These mice are on 129X1/SvJ background. The C1q knockout mice (C1q^-/-^) are on C57BL/6 background [14]. The two strains were interbred (to generation F8) in order to give rise to the two strains used in all experiments: mTTR^-/-^hTTR^Met30+/+^ mC1q^+/+^ (designated V30M) and mTTR^-/-^hTTR^Met30+/+^mC1q^-/-^ (designated V30M C1q KO), both on a mixed background of 129X1/SvJ / C57BL/6.

The age of the animals ranged from 3 to 18 months and examined in three groups; group A (3–6 months), group B (9–12 months) and group C (15–18 months). For the V30M mice, each age group was comprised of 10 mice (5M/5F), whereas for the V30M C1q KO groups, of 15 mice (7M/8F) each. All animals were kept in a regular 12-hour light-12 hour dark cycle and were given free access to water and food, under SPF conditions. Animals were separated in cages depending on the age group they were assigned to and their sex. All animal involving experiments were carried out in accordance to the 86/609/EEC Directive. Also, a project license was obtained from the Cyprus Veterinary Services approving the methodology (License Number: CY/EXP/PR.L3/2012)

Mice were anesthetized and then euthanized using Tribromoethanol (Avertin) through IP injection at a dose of 250 mg/Kg. The animals were then exsanguinated via PBS perfusion to reduce the contribution of plasma in tissue measurements. Tissues were obtained at the required time points. Tissues were processed for immunohistochemistry by carrying out overnight 4% PFA fixation followed by wax embedding or were frozen and kept at -80°C for western blot analysis. Amyloid deposition assessment was confined to stomach tissue since this tissue is heavily involved in amyloid deposition at an early age in this particular mouse model of ATTRV30M neuropathy.

### Transgene zygosity assessment

For transgene zygosity assessment, the qPCR LightCycler FastStart DNA Master SYBR Green I assay (Roche Cat. No. 12 239 264 001) was used according to the manufacturer’s instructions. The primers (same used for hATTRV30M genotyping) were used at 0.5μM concentration each. Wild type mice (without the transgene), heterozygote and homozygous mice were used as controls to allow for result interpertation. The GAPDH gene was used as a run control for normalisation (mGAPDH F 5’–CGACTTCAACAGCAACTCCCACTCTTCC– 3’ & mGAPDH R 5’ TGGGTGGTCCAGGGTTTCTTACTCCTT—3').

### Genotyping

Animals were routinely genotyped using the PCR method. Primers for the mouse TTR gene (mTTR F 5'—CTG ACC CAT TTC ACT GAC ATT T—3'& mTTR R 5'–CAA ATG GGA ACC TGG AAC C—3'); the human mutated transgene (hMET30 F 5’–TGCTGATGACACCTGGGAGC– 3’ & hMET30 R 5’ TCAGGTTCCTGGTCACTTCC—3') and for the C1q gene (mC1qA/5'+—GGG GCC TGT GAT CCA GAC AG, mC1qIN/2—TAA CCA TTG CCT CCA GGA TGG & Neo 3'—GGG GAT CGG CAA TAA AAA GAC) were utilized for screening with annealing temperature at 58°C.

### hTTR mRNA expression in liver tissues

Two-step RT-qPCR was carried out to quantify the expression of hTTR in the liver. Liver tissue was extracted following animal exsanguination via PBS perfusion. Approximately 25mg of liver tissue was removed and lysed using the RNeasy Mini QIAcube Kit (74116). RNA concentration was assessed by Nanodrop2000 and maximum 100ng/μl was used for cDNA synthesis. The Invitrogen SuperScript^™^ II Reverse Transcriptase (18064–022) was used to synthesize first-strand cDNA according to the manufacturer’s instructions.

TaqMan^®^ Gene Expression Assay for human TTR was then used, containing a pair of unlabelled PCR primers and a TaqMan^®^ probe with a FAM^™^ dye label on the 5' end, and minor groove binder (MGB) non-fluorescent quencher (NFQ) on the 3' end. 5μl 2x TaqMan^®^ Gene expression master mix was used with 0.5μl hTTR primer probe mix (Hs00174914_m1, 4331182) and 2μl cDNA in a total reaction of 10μl. The GAPDH (4352932E) gene was used as an endogenous control in delta Ct normalization of samples. For each liver sample duplicate reactions were run.

### Western blots and densitometry

**Fig 1:**
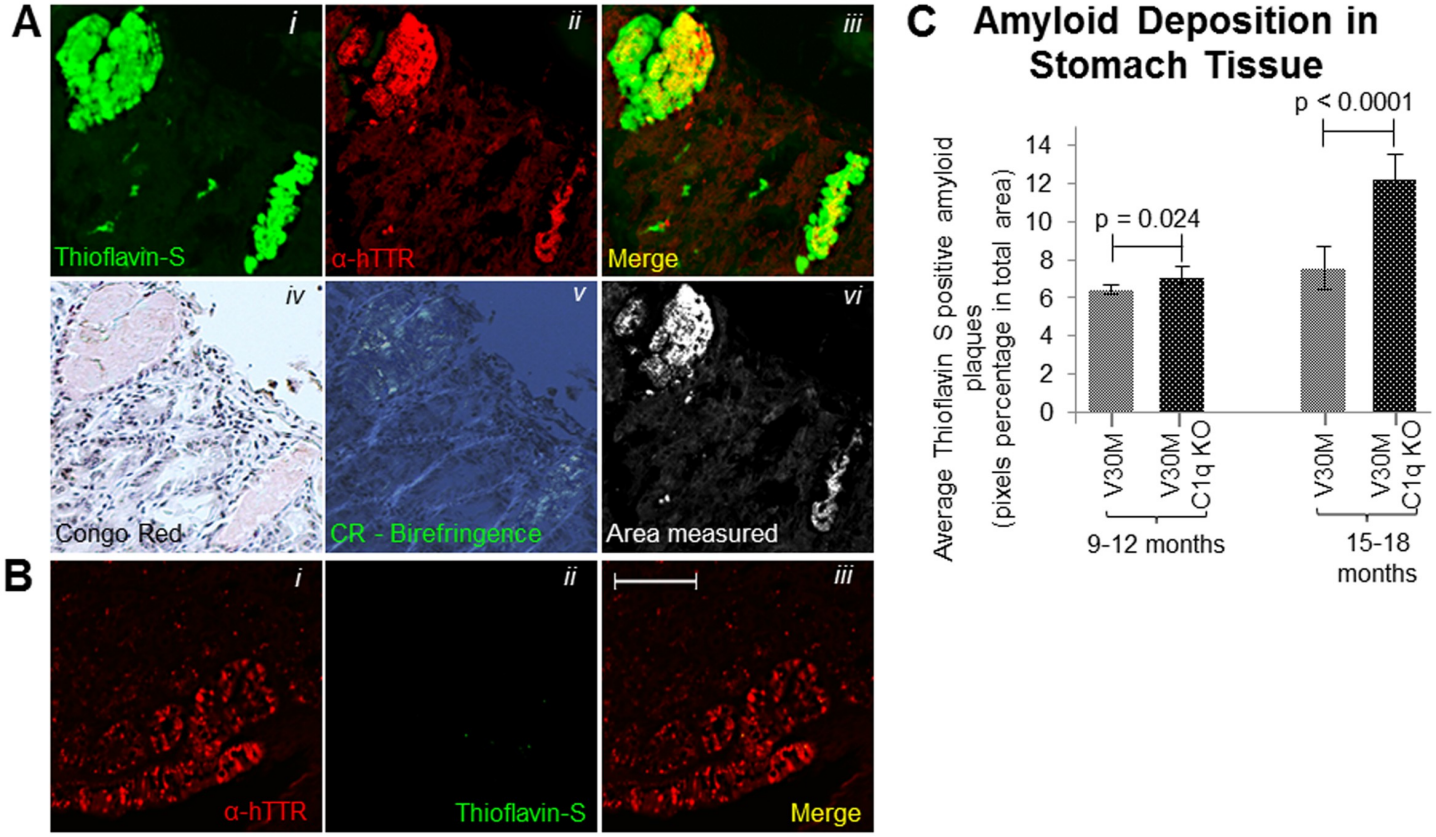
Amyloid deposition. Stomach tissue from 15 month V30M animal stained with Thioflavin-S, α-hTTR antibody and Congo red in consecutive sections (*Ai-Avi*). The area of Thioflavin-S and α-hTTR antibody co-localization which was included in measurements as TTR plaques is indicated in (*Avi*). Stomach tissue from 5 month V30M animal stained with stained with Thioflavin-S and α-hTTR antibody indicating solely the presence of pre-fibrillar hTTR (Bi-*Biii*) (*A&B* Scale bar = 75mm). *C* Quantification of the amyloid plaques found in the stomach measured through Thioflavin-S and α-hTTR antibody co-localization for both the V30M and V30M C1q KO mice (n = 15/age group/line). Data presented as mean ± 1SD.

Stomach homogenate (tissue lysed with RIPA buffer and protease inhibitors under sonication) was separated via reducing SDS-PAGE and transferred onto nitrocellulose membranes. The membranes were blocked with 5% low fat dry milk (Regilait) for one hour at room temperature. The membranes were then incubated overnight at 4°C with anti-rabbit Transthyretin (TTR, human) (DAKO A000202) at a dilution of 1/2,000. The antibody was visualized using the Amersham ECL Western Blotting Detection Reagent (RPN2232) after incubating with anti-rabbit HRP conjugated secondary antibody at 1/5,000 (Jackson ImmunoResearch 111-035-003) for one hour at room temperature. Blots were repeated in triplicates and were visualized using the UVP bio-imaging system. It should be noted that for TTR, due to the denaturing conditions, only monomers (14 KDa) were visualized although our ladder allowed visualization up to 210 KDa (Fig 1A). Therefore only hTTR monomers and not plaque-bound hTTR was measured since the latter needs to be otherwise extracted [15, 16].

Image J was used to carry out densitometry calculations, while all bands were normalized against a GAPDH loading control (Santa Cruz Biotechnology anti-mouse SC-32233 1/4,000 and anti-mouse HRP conjugated secondary antibody Jackson ImmunoResearch 115-035-003, 1/5,000).

Serum TTR was similarly measured by western blot in the blood of 5 animals from each of the two transgenic groups of mice. Blood samples were collected, without sacrificing any of the animals, from the orbital sinus in the absence of anticoagulant. The samples were allowed to stand overnight at 4°C to coagulate. They were then centrifuged at 3,500 rpm for 10 minutes and the top layer was collected in order to obtain the serum. Samples were diluted 1/10 using injection water prior to western blot analysis as aforementioned. However, the samples mixed with loading buffer were not boiled but were instead heated at 65°C for 15 minutes to avoid protein coagulation.

Further immunoblotting analysis was carried out to evaluate the expression of various other markers: Fas (Santa Cruz Biotechnology anti-rabbit sc-1023 1/1000) and activated Caspase-3 (Santa Cruz Biotechnology anti-goat sc-1225 1/500), CD68 Fitch conjugated (Abcam species anti-rat ab53444 1/400), GRP78 (Santa Cruz Biotechnology anti-rabbit sc-13968 1/1000),C5b-9 (EMD Millipore anti-rabbit 204903 1/4,000), Properdin (Santa Cruz Biotechnology anti-mouse sc-393723 1/500), C5a (Santa Cruz Biotechnology anti-goat sc-21941) and CD88 (Santa Cruz Biotechnology anti-mouse sc-53795). The appropriate HRP conjugated secondary antibodies were used, anti-mouse (Jackson ImmunoResearch 115-035-003, 1/5,000), anti-rabbit (Jackson ImmunoResearch 111-035-003) and anti-goat (Jackson ImmunoResearch 705-035-003, 1/5000).

### Amyloid plaque visualization and quantification

Thioflavin S stain combined with TTR immunofluorescence were used to identify TTR specific amyloid deposits in paraffin sections obtained from stomach tissue. Paraffin sections were deparaffinised and hydrated to distilled water. Sections were then stained with Mayer's haematoxylin for 5 minutes, washed further with distilled water and then stained with aqueous 1% Thioflavin S solution (Sigma Aldrich T1892-25G) for a further 5 minutes and finally differentiated in 50% ethanol before been rinsed with distilled water and then mounted using the DAKO Fluorescence Mounting Medium (S3023). Thioflavin S positive deposits were further confirmed to be amyloid by Congo Red (Fig 1A) and electron microscopy (data not shown). Plaques positive for both Thioflavin S and hTTR were measured using the Image J software set to measure yellow (570–585 nm) (Fig 1A*iii* and 1A*vi*). TTR amyloid plaques were measured over the entire area of stomach section, a percentage of the surface area occupied by plaques was calculated and an average percentage obtained over three serial sections.

### Immunohistochemistry for TTR and other molecular markers

Paraffin sections from animals’ stomachs were deparaffinised and hydrated to distilled water. Sections were then blocked with 5% BSA solution in PBS for 1 hour at room temperature and then incubated with the appropriate primary antibody overnight at 4°C. The primary antibody used was the anti-human Transthyretin (TTR) from DAKO (A000202) at a dilution of 1/500. The slides were then incubated with Invitrogen Alexa Fluor 555 fluorescence secondary antibody for 1 hour at room temperature at 1/2,000 (anti-rabbit A-21428). Finally, sections were washed with PBS and mounted using the DAKO Fluorescence Mounting Medium (S3023).

Further analysis was carried out using supplementary antibodies; Fas (Santa Cruz Biotechnology anti-rabbit sc-1023 1/1000) and activated Caspase-3 (Santa Cruz Biotechnology anti-goat sc-1225 1/500), CD68 Fitch conjugated (Abcam ab53444 1/100), GRP78 (Santa Cruz Biotechnology anti-rabbit sc-13968 1/1000), C5b-9 (EMD Millipore anti-rabbit 204903 1/4,000), Properdin (Santa Cruz Biotechnology anti-mouse sc-393723 1/500) and C1q (Santa Cruz Biotechnology anti-goat sc-27661 1/250). The appropriate Invitrogen Alexa Fluor 555 fluorescence secondary antibodies were used, anti-rabbit (A-21428 1/2000) and anti-goat (A-21432 1/2000).

### Morphometric image analysis

This analysis was carried out to measure areas of positivity by immunofluorescence among the various animals. Pictures were taken using a Zeiss fluorescence microscope using the 20X objective lens (for measurement purposes). Four (4) pictures were taken of each stomach in order to visualize the entire stomach cross section. Percentage area of antibody positivity was measured using the Image J software set to measure red (665-700nm) and averaged from three serial cross sections for each animal. A control (wild animal) was similarly measured to obtain an indication of background positivity.

### Statistical analyses

Using the Microsoft Office Excel 2010 suite, mean, standard deviation and p-values were calculated. Using this information, graphical charts representing the data were prepared.

## Results

### Amyloid deposition

Bearing in mind that the major hallmark of TTR amyloidosis is plaque formation, Thioflavin S staining along with hTTR immunohistochemistry (Fig 1A) were used to record and quantify TTR specific amyloid deposition among the three age groups of the V30M C1q KO mice and of the V30M mice expressing endogenous C1q.

Comparison between the two strains of mice has shown that there was no detectable amyloid in stomach tissue examined at 3–6 month of age although pre-fibrillar TTR is seen to be deposited (Fig 1B). Between 9 and 12 months, amyloid deposition was detected with a significant difference between the two strains (Fig 1C). The V30M C1q KO mice exhibited a more marked statistically significant increase amyloid by 15 to 18 months of age compared to the control V30M mice (Fig 1C).

### Transthyretin levels in the stomach and serum

**Fig 2:**
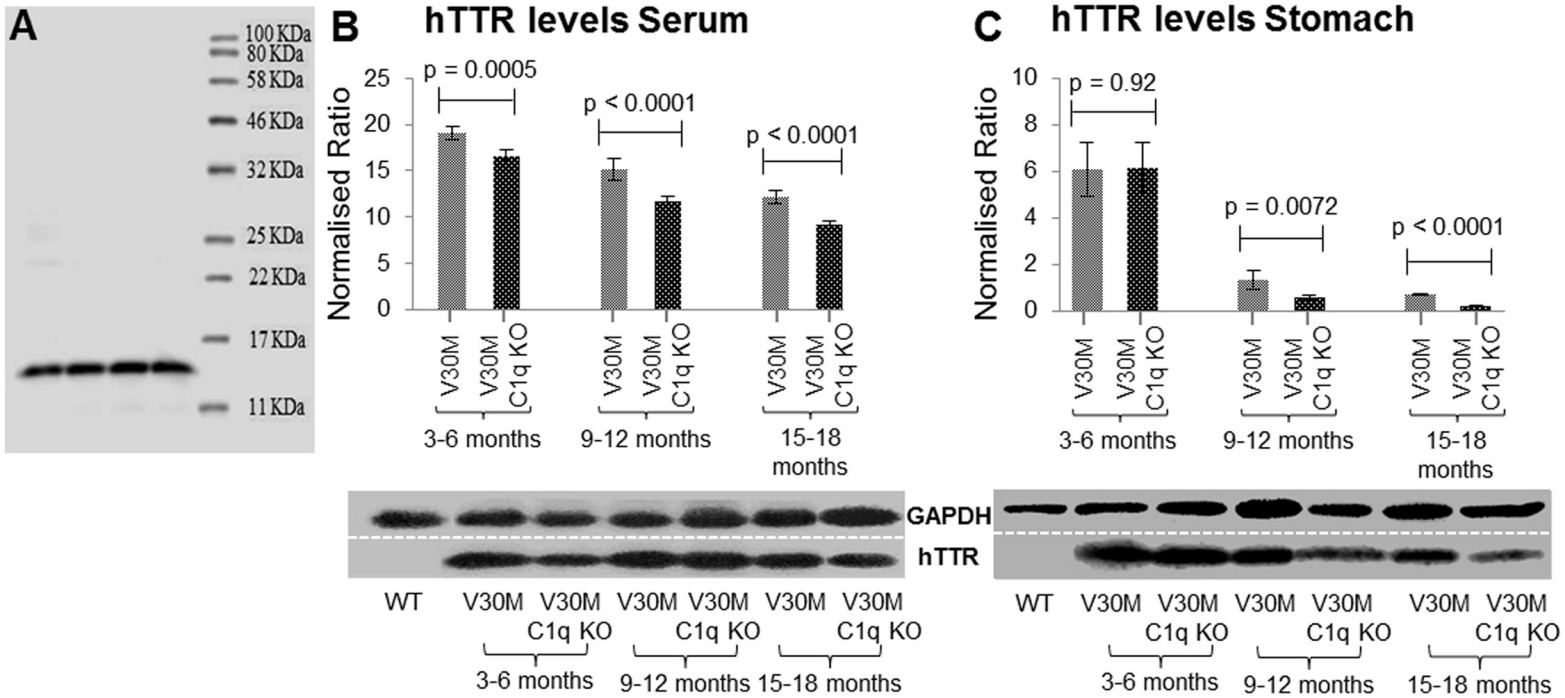
hTTR levels in serum and stomach. hTTR immunoblotting exhibiting a single band at 14KDa (*A*). hTTR levels measured via immunoblotting in serum of V30M and V30M C1q KO animals of all three age groups indicating higher hTTR in the V30M mice and a gradual decrease in the amount of hTTR present as the animals age (*B*). hTTR levels measured via immunoblotting in stomach tissue of the V30M and V30M C1q KO animals exhibit a severe decrease in the second age group (*C*). *B&C* n = 15/age group/line. Data presented as mean ± 1SD.

TTR is produced as a monomer which assembles into a tetramer before being secreted by the liver into the bloodstream. Previous studies have indicated that ATTRV30M patients have significantly less circulating TTR than their respective control group [17]. The instability of the mutant tetramer makes it more prone to dissociation into monomers which misfold and eventually get deposited in various tissues as amyloid [18]. The difference in the levels of the mutated hTTR in both the serum and the stomach tissue between the two strains of mice across all age groups was evaluated via immunoblotting. There was a significant decrease in the amount of circulating hTTR in serum with age in both strains but more so in V30M C1q KO mice (Fig 2B). Monomers from pre-fibrillar hTTR in stomach tissues were found markedly reduced between the first and second age groups coinciding with the appearance of significant Congo-red amyloid deposits in both strains (Fig 2C).

### Complement markers

Since the classical pathway was ablated there was no detectable amount of C1q found, either by immunoblotting or immunohistochemistry in the V30M C1q KO mice (data not shown). Markers for other components of the complement were used to assess the activation of complement.

**Fig 3:**
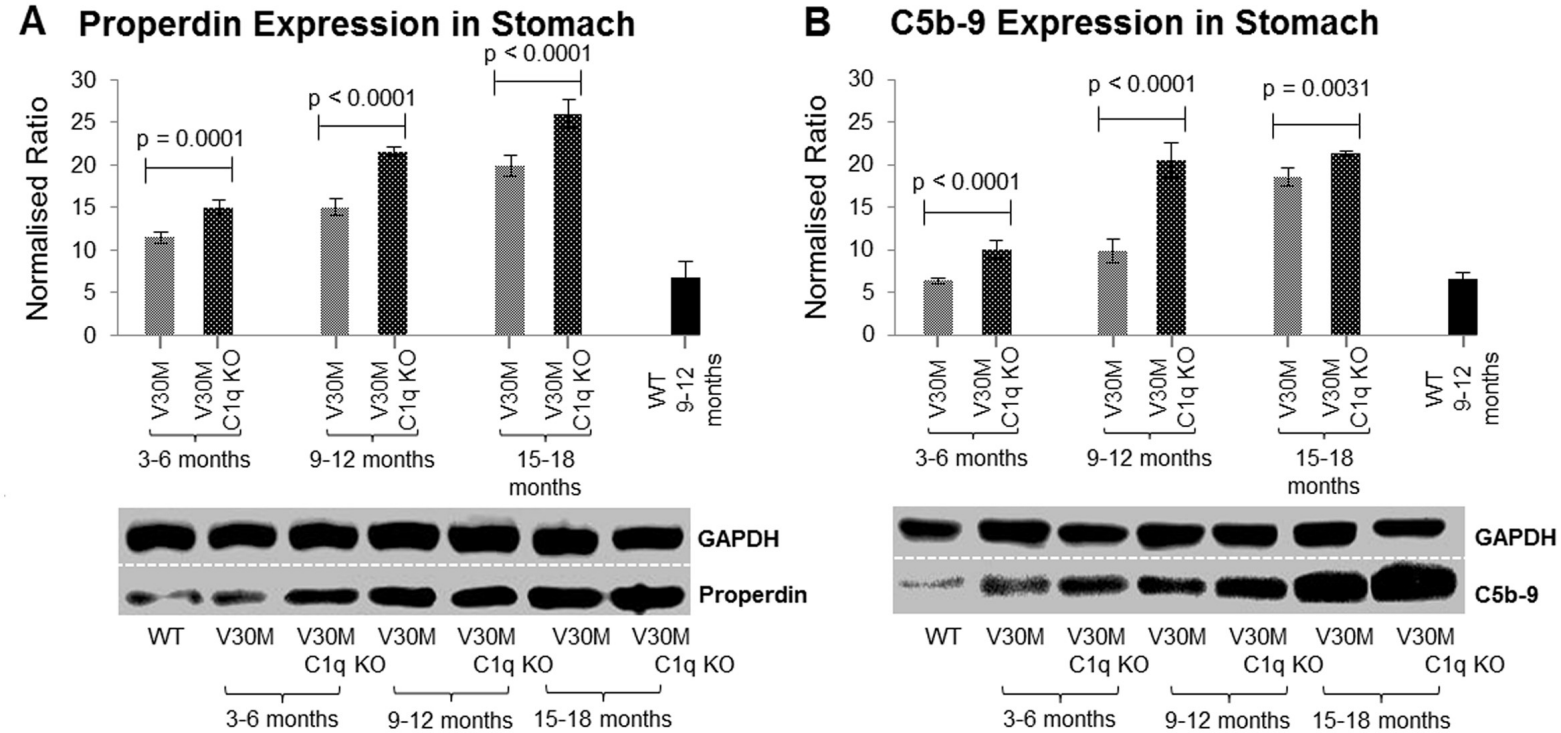
Expression of complement markers. The expression of Properdin (*A*) and C5b-9 (*B*) measured via immunoblotting in both V30M and V30M C1q KO animals stomach tissue in all three age groups exhibiting an age related increase of both markers, as well as a higher expression in the V30M C1q KO animals (n = 15/age group/line, data presented as mean ± 1SD).

Properdin serves as an activator of the alternative pathway as well as a stabilizer of the C3 convertases [19]. Properdin essentially acts as an indicator of the activation of the alternative pathway. Our data show that properdin, and thus the alternative pathway is up regulated in both the V30M and V30M C1q KO mice compared to control but more so the C1q deficient mice perhaps explaining the unexpectedly higher levels of C5b-9 in this strain (Fig 3A).

Activation of any of the three complement pathways ultimately leads to the formation of the C5b-9 terminal attack complex which promotes cell lysis as well as act as a pro-inflammatory agent [20]. Our results indicate that C5b-9 expression is up regulated in both strains compared to control but unexpectedly more so in V30M C1q KO mice despite the lack of activation via the classical pathway (Fig 3B).

### Apoptotic markers

**Fig 4:**
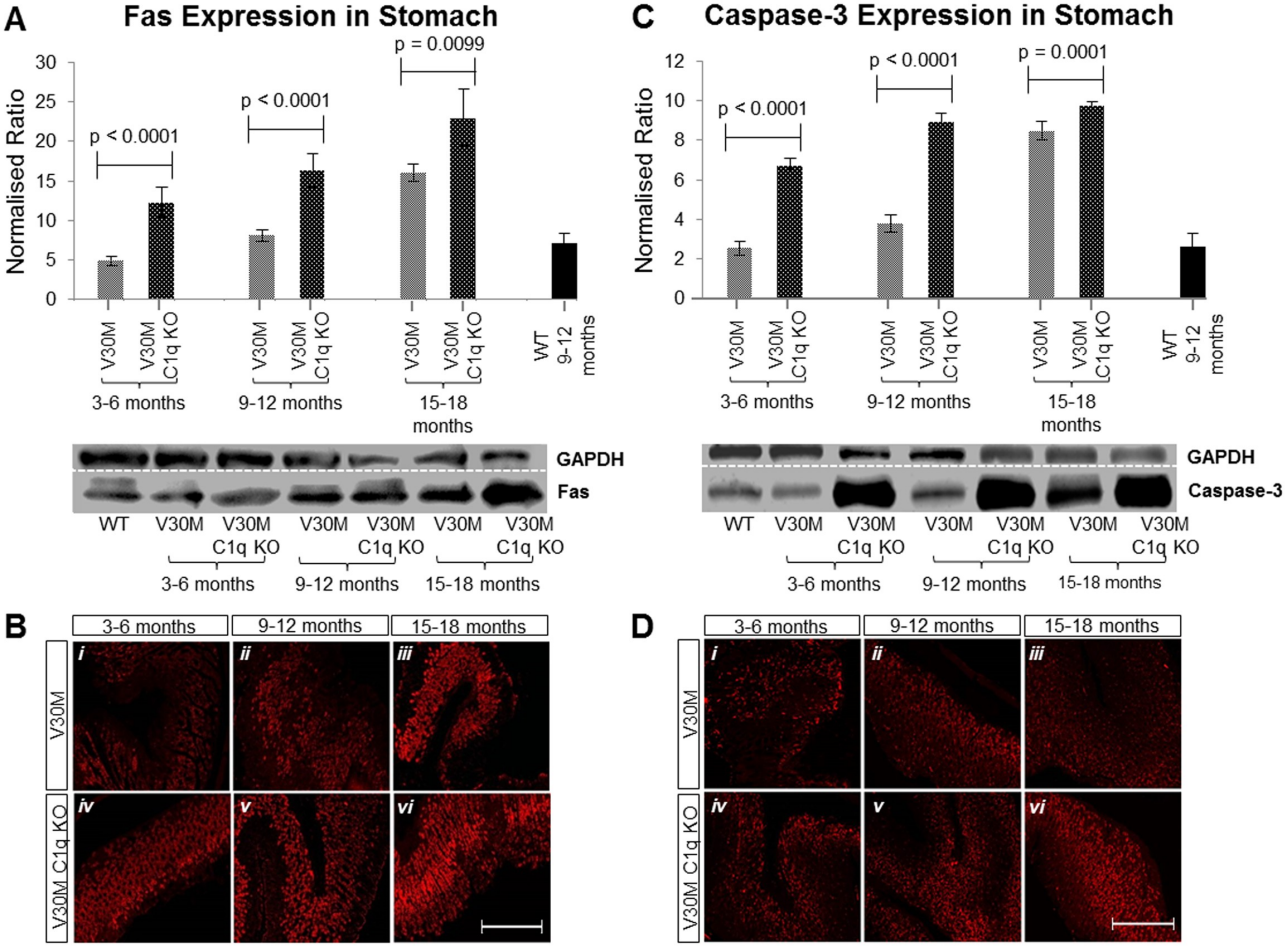
Expression of apoptotic markers. The expression of Fas protein measured by immunoblotting (*A*) in stomach tissue of both V30M and V30M C1q KO animals in all three age groups indicating an age related steady increase, as well as a higher expression in the V30M C1q KO mice. The same was observed by immunofluorescence (*B*). Similarly, the expression of Caspase-3 was also measured by immunoblotting (*C*) again indicating an age related increase with a markedly increased expression in the V30M C1q KO mice. Similar observations were made through immunofluorescence (*D*). A&C n = 15/age group/line, data presented as mean ± 1SD. *B&D* Scale bar = 150mm.

The Fas death-receptor is a member of the tumor necrosis factor receptor family known to trigger apoptosis [21]. The Fas death-receptor has been previously shown to be up regulated in V30M mice, probably as a result of increased apoptosis triggered by amyloid deposits [22]. When comparing the V30M C1q KO mice to V30M mice we noted a significant increase in the expression of the Fas death-receptor across all age groups consistent with the higher level of amyloid deposition observed in this strain (Fig 4A and 4B).

The levels of expression of caspase-3, one of the most prominent executioner caspases [23], were also measured. Previous studies have shown caspase-3 to be elevated in V30M mice even before mature fibril formation [18]. We also recorded a significant up regulation of caspase-3 in both V30M C1q KO and V30M mice, but more in the former, again consistent with the higher level of amyloid deposition observed in this strain (Fig 4C and 4D).

### Stress markers

**Fig 5:**
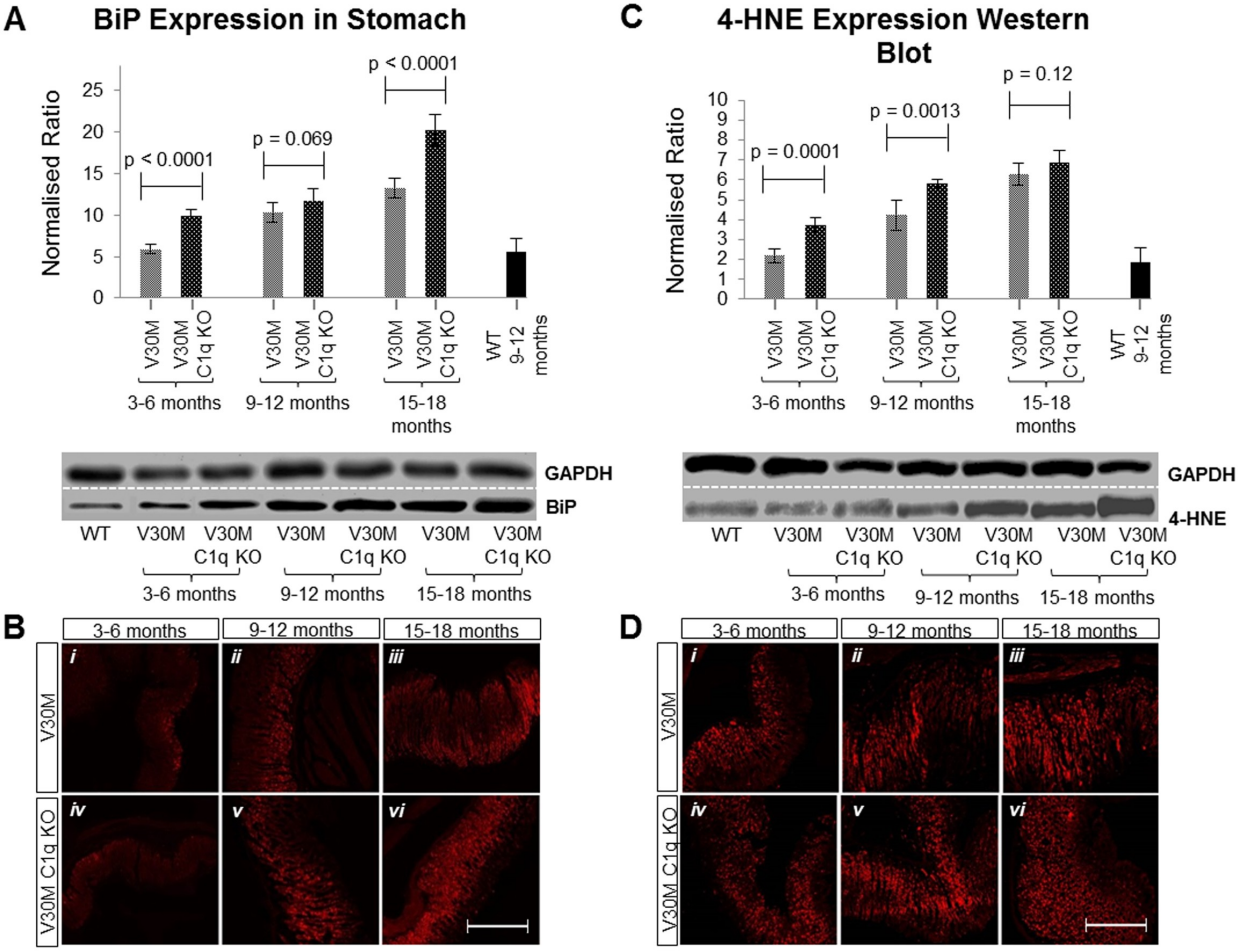
Expression of stress markers. The expression of BiP protein measured by immunoblotting (*A*) in stomach tissue of both V30M and V30M C1q KO animals in all three age groups depicting an increased expression in V30M C1q KO mice of all ages. The same was observed by immunofluorescence (*B*). Furthermore, the presence of the 4-HNE was quantified via immunoblotting (*C*) in stomach tissues of both V30M and V30M C1q KO animals in all three age groups, again indicating the higher expression of 4-HNE in V30M C1q KO mice. The same was observed by immunofluorescence (*D*). A&C n = 15/age group/line, data presented as mean ± 1SD. *B&D* Scale bar = 150mm.

The endoplasmic reticulum (ER) stress marker BiP (induced by the increase of misfolded proteins in the ER) [22] was also shown to be more increased in the V30M C1q KO mouse. The most substantial increase was observed during the third age group which also presented with the highest amount of amyloid deposition (Fig 5A and 5B).

In order to assess oxidative stress we used 4-hydoxynoneal (4-HNE), a by-product of lipid peroxidation. 4-HNE has been previously implicated in ATTRV30M neuropathy and found to be elevated in human amyloid tissues [24]. 4-HNE was increased in both strains compared to control but more so in the V30M C1q KO mice (Fig 5C and 5D).

### Macrophage marker

**Fig 6:**
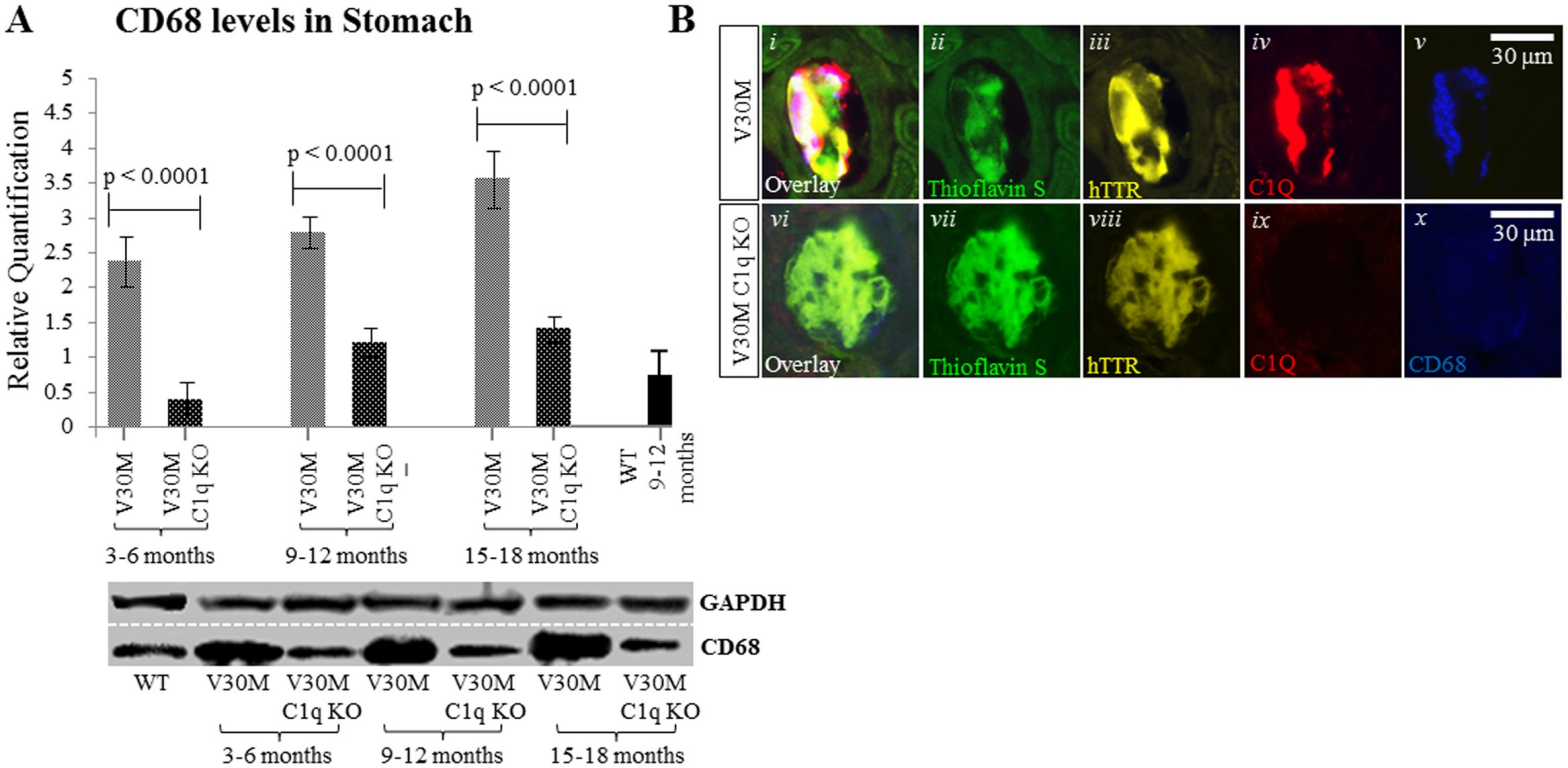
Expression of CD68. Immunofluorescence indicating the absence of the pan-macrophage marker CD68 on hTTR positive plaques from the V30M C1q KO mice contrary to V30M mice where macrophages co-localize with C1q (*A*) n = 15/age group/line, data presented as mean ± 1SD. By Western blot expression of the marker CD68 was severely decreased in stomach tissue of the V30M C1q KO mice when compared to the V30M mice (*B*), Scale bar = 30mm.

Macrophages have been shown to act as the final effectors in the process of amyloid removal [25]. Considering the role of C1q in opsonization and enhancement of phagocytosis as well as its effect on Mer Tyrosine Kinase expression [12, 26] it seemed appropriate to investigate the effect of C1q ablation on macrophages. The widely used CD68 generic macrophage marker was used in double staining with an anti-C1q antibody. Serial sections were also stained with Thioflavin S and anti-hTTR antibody. In the V30M mice there was co-localization of C1q and CD68 positivity with hTTR specific amyloid plaques consistent with facilitation of macrophage mediated phagocytosis by C1q (Fig 6B*i*). In C1q deficient mice however there is no expression of either CD68 or C1q positivity on amyloid plaques (Fig 6B*vi*), suggesting that C1q may be required for effective macrophage recruitment. This visual interpretation was supported by Western blot data (Fig 6A).

### C5a anaphylatoxin and receptor markers

The complement component 5a (C5a) is a product of the cleavage of complement factor 5 (C5). C5a is a potent anaphylatoxin involved in inflammatory response cell chemotaxis, as well as cytokine and chemokine release and most importantly phagocytosis [27, 28]. The receptor for C5a, CD88 is expressed amongst a variety of peripheral cells, including neutrophils and monocytes [29]. C5a is heavily associated with innate immunity based on its chemotactic function in recruiting inflammatory cells such as neutrophils and macrophages, as well as activating phagocytic cells and oxidant generation [27]. In view of the observed reduction of CD68 positive cells in the C1q deficient mice, the expression of the C5a anaphylatoxin and its receptor CD88 (C5aR) were analyzed using immunoblotting.

**Fig 7:**
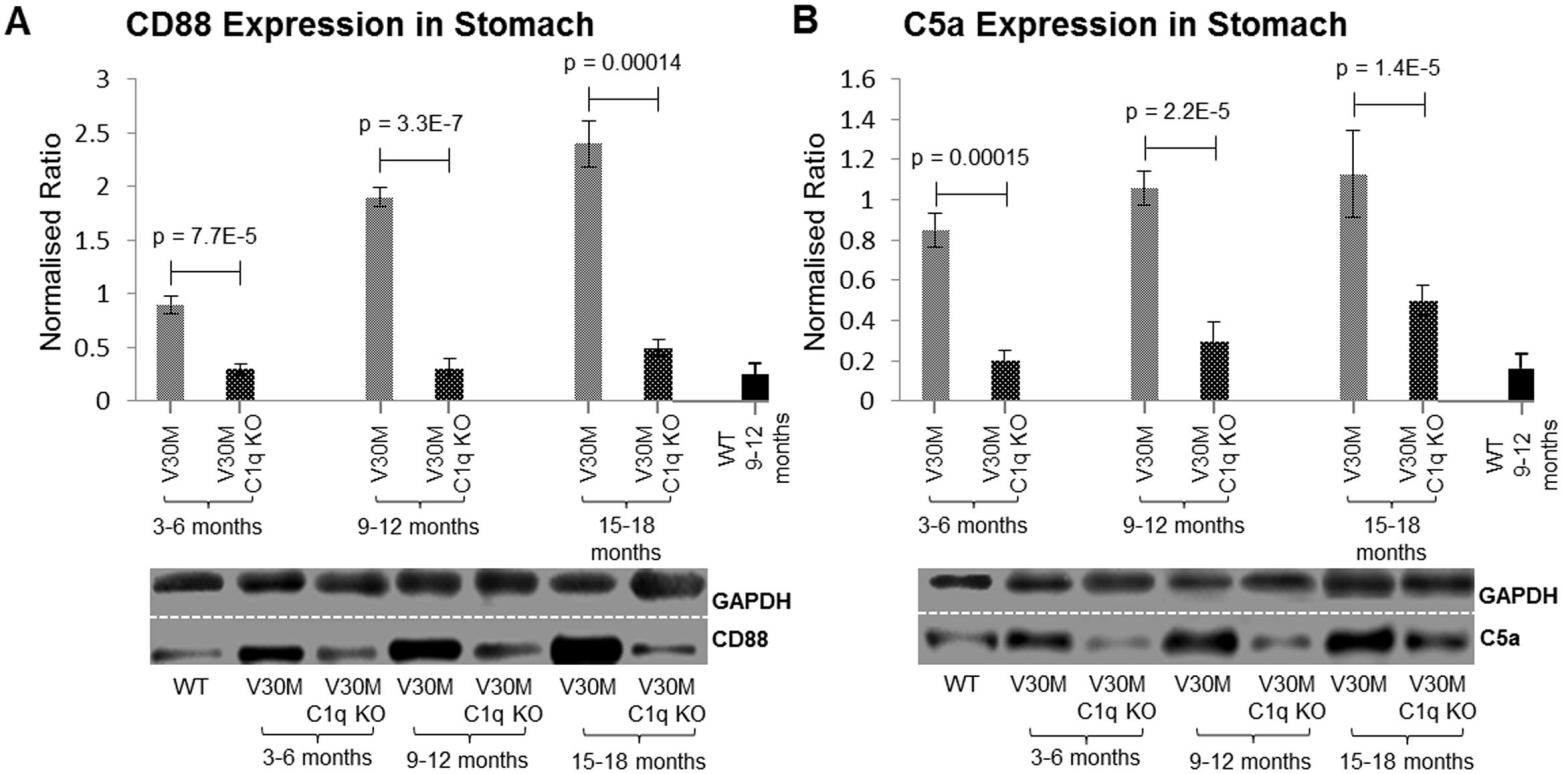
Expression of CD88 and C5a. The expression of the CD88 receptor (*A*) and its ligand C5a (*B*) were measured by immunoblotting in stomach tissues of both V30M and V30M C1q KO animals in all three age groups. Both markers exhibit an astounding reduction in the V30M C1q KO mice. A&B n = 15/age group/line, data presented as mean ± 1SD.

Our results indicate that both the expression of the CD88 receptor molecule (Fig 7A) and its ligand, C5a (Fig 7B) are markedly decreased in the V30M C1q KO mice when compared to the V30M mice in all three age groups.

## Discussion

Stimulation and eventual activation of any of the complement pathways is an integral part of the inflammatory response not only in response to injury but also in the removal of invading foreign material including amyloid. Furthermore, the complement system is vital in clearing up apoptotic cells, cellular debris and protein aggregates. On the downside, activation of the complement cascade has been associated with pathogenic pathways in several neurological conditions. In multiple sclerosis, complement activation has been linked to oligodendrocyte damage and demyelination [30, 31]. In Alzheimer’s disease activated components of the classical complement pathway, such as C1q and C5b-9, have been suggested to promote neuroinflammation as well as to be associated with amyloid plaques [32, 33]. C1q has been shown to increase fibrillogenesis in an Alzheimer’s double transgenic mouse model (APP+PS1) where injection of human C1q into the cortex and hippocampus increased Congo red-positive deposits [34]. However there is also evidence suggesting that C1q may actually act as a neuroprotective agent in AD, where addition of C1q in vitro provided neural protection, not via inhibition of SAP binding to neurons, but probably through another cellular mechanism [11]. Data concerning the effects of the complement on TTR amyloidosis however is scarce.

The exact relationship between C1q and amyloidogenesis is poorly understood as suggested by the contradictory evidence found in the existing literature, and its role may differ depending on tissue bed and the type of the amyloidotic peptide. A previous study in a complement deficient Alzheimer mouse model indicated that the levels of amyloid deposition between the C1q deficient model and the control strain were comparable with no detected significant difference [10]. Our results however show that as the V30M C1q KO mice get older amyloid deposition is significantly increased by over 60% compared to the complement efficient V30M strain (Fig 1C).

Prefibrillar TTR in stomach tissue was reduced, in both C1q efficient and deficient animals, in the 9–12 month age group which coincides with the sequestration of prefibrillar TTR into amyloid plaques. Serum TTR is decreased in both strains but more so in the C1q deficient animals due to greater sequestration of TTR into amyloid as demonstrated.

As expected, apoptotic and cellular stress markers were correspondingly also increased. Fas and Caspase-3 were found to increase significantly throughout all three age groups in both strains compared to control but especially in the C1q deficient mice when analyzed both by immunoblotting and by immunohistochemistry (Fig 4). Fas is a member of the tumor necrosis factor family, it is a potent inducer of apoptosis and forms the death-inducing signaling complex upon binding of the ligand to its receptor [35]. Caspase-3, known to be responsible not only for the regulated cleavage of death substrates but also the actual execution of apoptosis has been heavily implicated in ATTRV30M pathology (Sousa et al., 2001).

BiP (GRP78) acts as a “detector” of endoplasmic reticulum (ER) stress, setting in motion the unfolded protein response when activated. Increased levels of BiP have been reported in biopsies of ATTRV30M patients and in transgenic mouse models [22].

Complement markers, C5b-9 and properdin (Fig 3) were measured to evaluate the effect of C1q deficiency on the membrane attack complex (MAC) formation as well as alternative complement pathway activation respectively. Properdin is a positive effector and regulator of the alternative pathway which also stabilizes the alternative pathway convertases (C3bBb) [36]. Previous studies have shown that C1q itself has the ability to bind C3b and essentially inhibit the activation of the alternative pathway [37]. Thus, lifting C1q inhibition on C3b would be expected to facilitate alternative pathway activation which due to the “tick over” effect is essentially always active due to the spontaneous hydrolysis of C3 [38].

Properdin has also been detected in Alzheimer transgenic mice amyloid plaques; indicating that activation of the alternative pathway probably also occurs in Alzheimer’s disease [39]. Our results indicate a continual increase in properdin expression amongst all age groups, particularly in the absence of C1q. The alternative pathway has also been found to be extremely more efficient in producing the terminal MAC when compared to the classical pathway by as much as fifteen times [40].

The results presented here suggest that loss of C1q results in less efficient opsonization/activation of macrophages during amyloid phagocytosis. C1q has been shown to be involved in complement-cascade-independent phagocytosis through monocytes and macrophages, more than 20 years ago; the detailed mechanisms however have not been fully elucidated yet [41, 42]. There is evidence that when immune complexes are bound by C1q they are more readily phagocytosed [43]. There is also data implicating C1q as the direct link between phagocytes and apoptotic cells, ultimately facilitating phagocytosis [26]. Studies attempting to isolate the complement component largely responsible for clearance of apoptotic cells have singled out C1q specifically since mice deficient of this component exhibited an enormous defect in phagocytosis of apoptotic cells [14, 44]. In order to further explore the contribution of the C3 and the alternative pathway in the clearance of apoptotic cells, peritoneal macrophages were studied in three distinct knockout strains of mice (C1qa^−/−^, C4^−/−^, C3^−/−^) where again only the C1q complex deficient strain exhibited a defect in clearing apoptotic cells through the resident macrophages [45]. In conjunction with studies reporting the existence of several possible C1q receptors on macrophages [46], as well as the molecule’s association with CR1[47], and the novel pathway through which C1q can trigger clearance of apoptotic cells by regulating the expression of Mer Tyrosine Kinase [12], the importance of C1q independent of the complement cascade seems likely. We have obtained immunohistochemical data displaying the co-expression of C1q and CD68 (a widely used macrophage marker) on the amyloid plaques (marked by double Thioflavin-S—hTTR positivity) in V30M mice. However, when analyzing the V30M C1q KO mice, the absence of C1q is also marked by the absence of CD68 (Fig 6B).

Apart from the role of C1q in macrophage recruitment independently of the complement pathway, activation of the classical cascade leads to the generation of the potent anaphylatoxin C5a. Both the anaphylatoxin and its receptor, CD88, have been observed to decrease severely in C1q deficient mice. Cleavage of C5 yields the anaphylatoxin C5a and C5b which is the first member in the assembly of the MAC. Even though reports have demonstrated direct cleavage of C5, independent of the complement pathway through other convertases [48–50], the release of C5a without the simultaneous formation of C5b-9 has never been shown [51]. Activation of both the classical and alternative pathways results in the generation of both effector molecules (36). Our data suggest that ablation of the classical pathway not only results in the up-regulation of the alternative pathway, but also results in the increase of the terminal MAC, while at the same time the amount of C5a detected is severely diminished in C1q ablated mice. Why the discordant result? Existing literature demonstrates that fluid-phase MAC has a half-life of 50 minutes whereas fluid-phase C5a can only be sustained for less than 2 minutes before been cleared from the circulation [52–54]. Considering that both C5a and C5b should be formed in equal quantities the total amount of MAC should reflect the total amount of C5a, either cell bound or fluid-phase [55]. Due to the short half-life of C5a accurate detection of the anaphylatoxin needs to take into account the amount produced, cleared away or bound by receptors [28]. Therefore the low levels of C5a in C1q ablated mice is probably accounted for by swift clearance of C5a in the absence of phagocytic cells which express the majority of C5a receptors and bind C5a.

**Fig 8:**
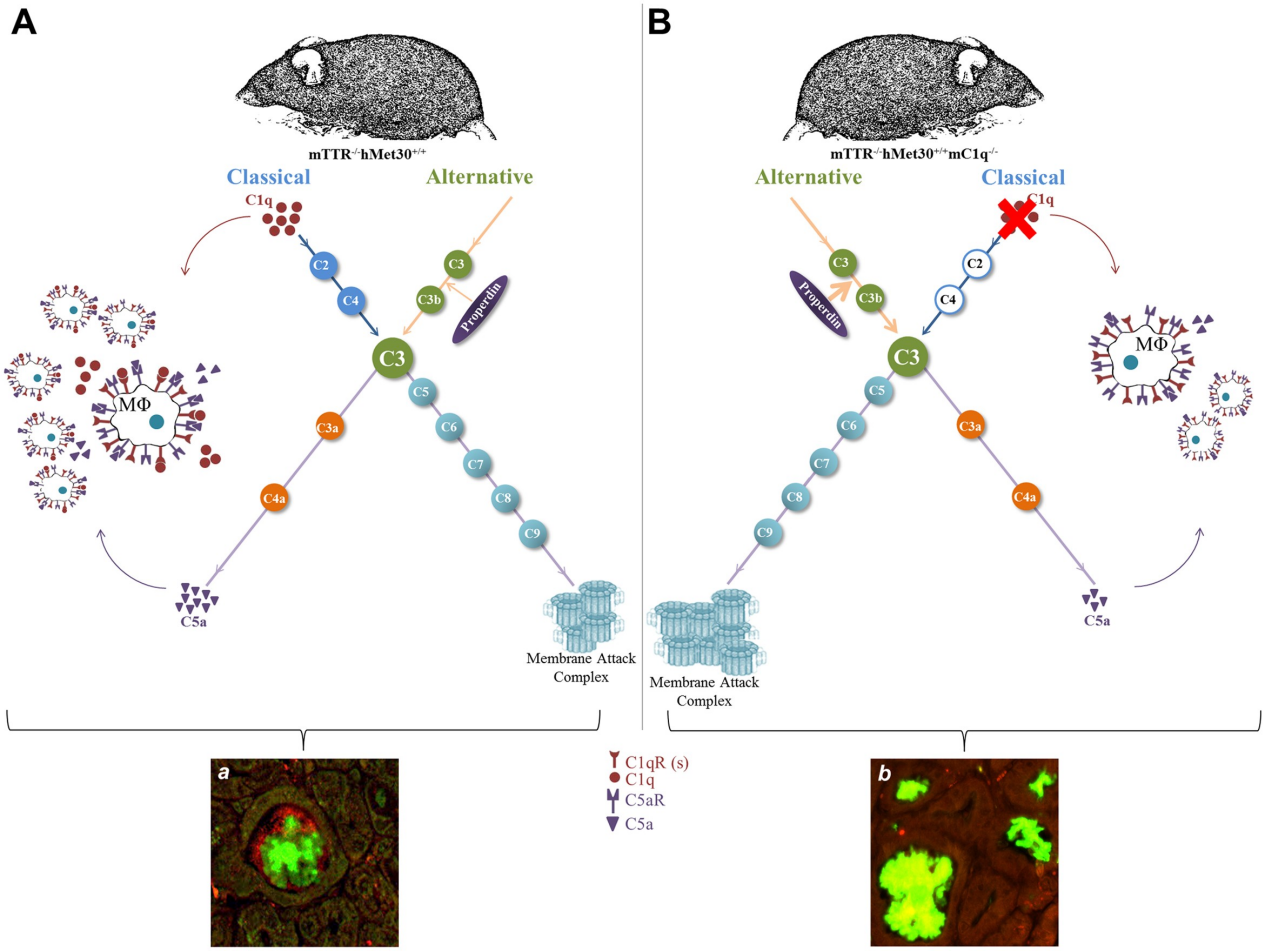
Model of observed C1q ablation effects. Classical and alternative pathway activation lead to the hydrolysis of C3 initiating the formation of the membrane attack complex (MAC) and production of the chemoattractant C5a. C1q and C5a recruit and activate phagocytic cells such as macrophages through receptors located on their surfaces (*A*). In the absence of C1q, properdin expression increases along with the entire alternative pathway and the terminal MAC complex. Concurrently, the presence of CD68 positive phagocytes was decreased along with the expression of C5a anaphylatoxin and its receptor CD88 (*B*). As a result, amyloid deposition increases following C1q ablation (*b*) versus the original transgenic model (*a*).

We propose that reduced recruitment of macrophages surrounding TTR plaques could explain the apparent increase in amyloid deposition and stress markers, indicating that the absence of C1q not only facilitates activation of the alternative pathway but also diminishes macrophage recruitment and thus their ability to clear up amyloid deposits. We have outlined these observations in the model depicted in Fig 8. These observations are in line with the findings of Bodin *et al*., which demonstrate the presence of complement mediated recruitment of macrophages when anti-SAP antibodies are injected in their model of amyloidosis [25]. Clearly the role of C1q and the complement cascade in general in the pathogenesis of hereditary ATTRV30M amyloidosis remains to be further elucidated. However it appears that C1q deficiency exacerbates amyloid deposition and supports the hypothesis that C1q is a modifier in ATTRV30M amyloid neuropathy.
